# Depressive symptoms are correlated with periaqueductal gray matter functional connectivity in migraine

**DOI:** 10.1192/j.eurpsy.2022.1636

**Published:** 2022-09-01

**Authors:** K. Gecse, D. Dobos, N. Károlyi, D. Baksa, C.S. Aranyi, M. Emri, G. Kökönyei, G. Bagdy, G. Juhasz

**Affiliations:** 1 Semmelweis University, Department Of Pharmacodynamics, Faculty Of Pharmacy, Budapest, Hungary; 2 Semmelweis University, Se-nap2 Genetic Brain Imaging Migraine Research Group, Budapest, Hungary; 3 University of Debrecen, Division Of Nuclear Medicine And Translational Imaging, Department Of Medical Imaging, Faculty Of Medicine, Debrecen, Hungary; 4 ELTE Eötvös Loránd University, Institute Of Psychology, Budapest, Hungary; 5 NAP-2-SE New Antidepressant Target Research Group, Hungarian Brain Research Program, Semmelweis University, Budapest, Hungary

**Keywords:** fMRI, migraine, depressive symptoms, PAG

## Abstract

**Introduction:**

Depression is the most common comorbidity of migraine. The brain of migraineurs with depression shows differences compared to migraine only or depression only patients. The comorbidity may affect specific regions such as the periaqueductal gray matter (PAG) which is important in negative emotion regulation and pain modulatory system.

**Objectives:**

We hypothesized that the alterations in PAG functional connectivity (FC) may play a role in migraineurs vulnerability for depression.

**Methods:**

A resting-state fMRI was conducted with 34 episodic migraine without aura patients and 41 control subjects. All participants were medication free and they did not have any psychiatric or chronic disorders. Depressive symptoms were measured with Zung Self-Rating Depression Scale. To investigate the relationship between depressive symptoms and PAG functional connectivity, Zung scores were used as covariates in each groups’ PAG-FC analysis using the Statistical Parametric Mapping (SPM12) toolbox in MATLAB environment.

**Results:**

There were no significant difference between migraine and control group in Zung scores (p=0.394). Negative correlation was found between Zung scores and PAG-FC with thalamus, fusiform gyrus, middle occipital gyrus and calcarine (p_FWE_<0.05) in migraine group. However, there was no significant correlation between Zung scores and PAG-FC in healthy control group.

**Conclusions:**

Our results suggest that PAG-FC with emotion and pain processing areas is affected by depressive symptoms in migraine patients, but not in healthy controls. Migraine patients without comorbid depression might have vulnerable neuronal pathways for depressive symptoms. A follow-up of these patients could be interesting to determine whether these connectivity alterations predict the possible comorbid depression.

**Disclosure:**

Hungarian Brain Research Program (2017-1.2.1-NKP-2017-00002, KTIA_13_NAPA-II/14, KTIA_NAP_13-1-2013- 0001, KTIA_NAP_13-2- 2015-0001); 2020-4.1.1.-TKP2020; ERA PerMed (2019-2.1.7-ERA-NET-2020-00005); ÚNKP-21-4-I-SE-15 (DB).

